# What is the Diagnostic Accuracy of Novel Urine Biomarkers for Urinary Tract Infection?

**DOI:** 10.1177/11772719221144459

**Published:** 2023-01-23

**Authors:** George Edwards, Anna Seeley, Adam Carter, Maia Patrick Smith, Elizabeth LA Cross, Kathryn Hughes, Ann Van den Bruel, Martin J Llewelyn, Jan Y Verbakel, Gail Hayward

**Affiliations:** 1 NIHR Community Healthcare Medtech and IVD Cooperative, Nuffield Department of Primary Care Health Sciences, University of Oxford, Oxford, UK; 2 Nuffield Department of Primary Care Health Sciences, University of Oxford, Oxford, UK; 3 Department of Global Health and Infection, Brighton and Sussex Medical School, Falmer, UK; 4 PRIME Centre Wales, Division of Population Medicine, Cardiff University, Cardiff, UK; 5 EPI-Centre, Academic Centre for General Practice, KU Leuven, Leuven, Belgium

**Keywords:** Systematic review, biomarkers, infection diagnosis, UTI

## Abstract

**Background:**

Urinary tract infection (UTI) affects half of women at least once in their lifetime. Current diagnosis involves urinary dipstick and urine culture, yet both methods have modest diagnostic accuracy, and cannot support decision-making in patient populations with high prevalence of asymptomatic bacteriuria, such as older adults. Detecting biomarkers of host response in the urine of hosts has the potential to improve diagnosis.

**Objectives:**

To synthesise the evidence of the diagnostic accuracy of novel biomarkers for UTI, and of their ability to differentiate UTI from asymptomatic bacteriuria.

**Design:**

A systematic review.

**Data Sources and Methods:**

We searched MEDLINE, EMBASE, CINAHL and Web of Science for studies of novel biomarkers for the diagnosis of UTI. We excluded studies assessing biomarkers included in urine dipsticks as these have been well described previously. We included studies of adult patients (≥16 years) with a suspected or confirmed urinary tract infection using microscopy and culture as the reference standard. We excluded studies using clinical signs and symptoms, or urine dipstick only as a reference standard. Quality appraisal was performed using QUADAS-2. We summarised our data using point estimates and data accuracy statistics.

**Results:**

We included 37 studies on 4009 adults measuring 66 biomarkers. Study quality was limited by case-control design and study size; only 4 included studies had a prospective cohort design. IL-6 and IL-8 were the most studied biomarkers. We found plausible evidence to suggest that IL-8, IL-6, GRO-a, sTNF-1, sTNF-2 and MCR may benefit from more rigorous evaluation of their potential diagnostic value for UTI.

**Conclusions:**

There is insufficient evidence to recommend the use of any novel biomarker for UTI diagnosis at present. Further evaluation of the more promising candidates, is needed before they can be recommended for clinical use.

## Background

Urinary tract infections (UTIs) are common across all age ranges and healthcare settings, with a lifetime risk of 50% to 60%.^1^ They are amongst the most common indications for antibiotics in the USA,^2^ and in England^3^ where they cost the NHS in the UK £316 million annually in emergency admissions for older adults alone.^4^ Over-treatment of suspected UTI and unnecessary treatment of asymptomatic bacteriuria (ASB) drive antibiotic overuse, and selection for antimicrobial resistance.^5,6^ Receipt of antibiotics for UTI results in carriage of resistant bacteria, which may persist for up to 12 months,^7^ and treatment of ASB increases the risk of recurrent infection.^8^ Thus current antimicrobial guidelines support prompt but targetted antibiotic prescribing, especially for older, multimorbid or frail patients, reliant on timely and accurate diagnosis of infection.^9^

Diagnosis of UTI is traditionally based on presence of typical symptoms, positive urine dipstick and growth of uropathogenic bacteria on urine culture.^10^ Each of these components presents problems. Many patients at risk of UTI may not experience typical symptoms, especially older adults, or those living in residential homes, where 40% of UTIs are incorrectly diagnosed.^11^ Even in younger women with uncomplicated UTI, the specificity of ‘typical’ symptoms such as dysuria, frequency and urgency is low, ranging from 20% to 45%.^12^

Urine dipstick tests to detect nitrates (presence of bacteria) and leucocytes are quick, simple and readily available across community and hospital settings. Nitrates have moderate specificity for infection (85%-98%)^13^ but are insufficient to rule-out UTI with a sensitivity of 30% to 40% . Presence of leucocyte-esterase is only modestly improves post-test probability of UTI, and is not diagnostic of infection in isolation.^1,4^ Due to the prevalence of ASB, urine dipsticks are unreliable in both older adults and catheterised patients.^14^ Accordingly, though widely used, results are commonly discounted in routine clinical practice.^15^

There are, too, limitations, in the diagnostic performance of urinary culture. Up to a third of urine cultures are contaminated by skin and/or faecal flora introduced during sampling^16^ and this has the potential to both obscure a true infection and give a positive culture result in the absence of infection. Further, cultures typically take 24 to 72 hours to report so it is usually necessary to make an antibiotic prescribing decision before culture results are available and they cannot distinguish infection from asymptomatic bacteriuria, especially in the elderly.^17^

Urine biomarkers could aid accuracy of UTI diagnosis and be developed into cheap, rapid point of care tests (POCTs), useful in settings without ready access to laboratory facilities. The last systematic review of urine biomarkers (in 2009) identified interleukins, notably IL-6 and IL-8, as potential candidates, but these had only been evaluated in a small number of studies.^18^ Over the last decade there has been a rapid expansion in biomarker technologies but there is insufficient evidence as to how these perform. In this review we therefore aimed to synthesise evidence of urine biomarkers for the diagnosis of UTI. We primarily used urine culture as our reference standard as this is widely used and easily comparable between studies. As a secondary aim we explored how urine biomarkers can distinguish UTI from ASB, given urine culture cannot differentiate these 2 conditions.

## Methods

Our review protocol was registered with PROSPERO in November 2019: CRD42019156071.

### Search

We searched Medline, Embase, CINAHL and Web of Science from inception until 11th April 2022 for studies with combined Medical Subject Headings and free text search terms in 3 main themes: urinary tract infection (eg, cystitis, UTI, bacteriuria); biomarkers (eg, biomarkers, immunoglobulins); and urine testing (eg, urinalysis, urine*, test*). The full search strategy is available in Supplemental Table 1.

### Eligibility

*Participants*: We included studies of adult patients (⩾16 years) with a suspected or confirmed urinary tract infection (including cystitis and pyelonephritis) or bacteriuria. We excluded studies not specifying the ages of included patients, of children under the age of 16, or where data for any patients under the age of 16 could not be disaggregated.

*Index tests*: We included studies of urine biomarkers. We considered a biomarker to be any substance which can be measured in the urine which may be indicative of medical state.^19^ This may arise through a biological or pathogenic process and includes markers of immune response and bacterial activity. This does not include detection of bacteria. We excluded studies of leucocyte esterase and nitrites, as they have been thoroughly studied, have modest test accuracy,^1,13,14^ and are insufficient for making a final diagnosis in clinical practice.^14,15,20^

*Reference standard*: We included studies with microscopy and/or culture as a reference standard and we did not specify a threshold for infection after culture as our aim was to offer a wide perspective on the available evidence for novel biomarkers. There is not one agreed threshold level of bacteria for diagnosing all UTIs^21,22^ and microscopy only can be useful for ruling out bacteriuria.^23^ We excluded studies using clinical signs and symptoms or dipstick only.

*Types of studies*: We included prospective cohort studies assessing diagnostic accuracy, cross-sectional studies, and case-control studies with a healthy control group. Although case-control studies risk exaggerating the differences between groups, by excluding cases for which diagnosis is difficult or unclear, and overestimate prevalence (spectrum bias), we included studies with this design as we did not expect to find a large number of cohort studies.

*Settings*: We did not exclude studies based on their clinical setting.

### Selection of studies

The Cochrane Collaboration Covidence platform was used for study screening.^24^ Two authors (GE, GH, ELAC, KH, AVDB, JV, MPS, AES, AC) screened each study according to prespecified inclusion and exclusion criteria and we resolved disagreements by discussion with a third reviewer. We screened titles and abstracts initially and obtained full texts for potentially relevant studies. We hand searched reference lists in relevant systematic reviews for relevant studies.

### Data extraction

We extracted study information, participant characteristics, index test description and process, statistical analysis, and results using a data extraction form designed by GE and piloted by GE, KH and MPS. One author (GE, AC, MPS, KH, AES) chosen at random performed data extraction using a standardised and piloted data extraction form. This was checked by a second author, chosen by availability, for accuracy.

### Risk of bias assessment

One author (GE, AC, MPS, KH, AES) assessed the risk of bias in the procedures of each included study using an the QUADAS-2 framework.^25^ This was checked by a second reviewer and any disagreements were resolved through discussion. One author reviewed the appraisal of each included study for consistency.

### Analysis

Due to study heterogeneity and a paucity of diagnostic accuracy data we were unable to perform meta-analyses as initially intended. We have summarised our results narratively.

## Results

**Figure 1.**
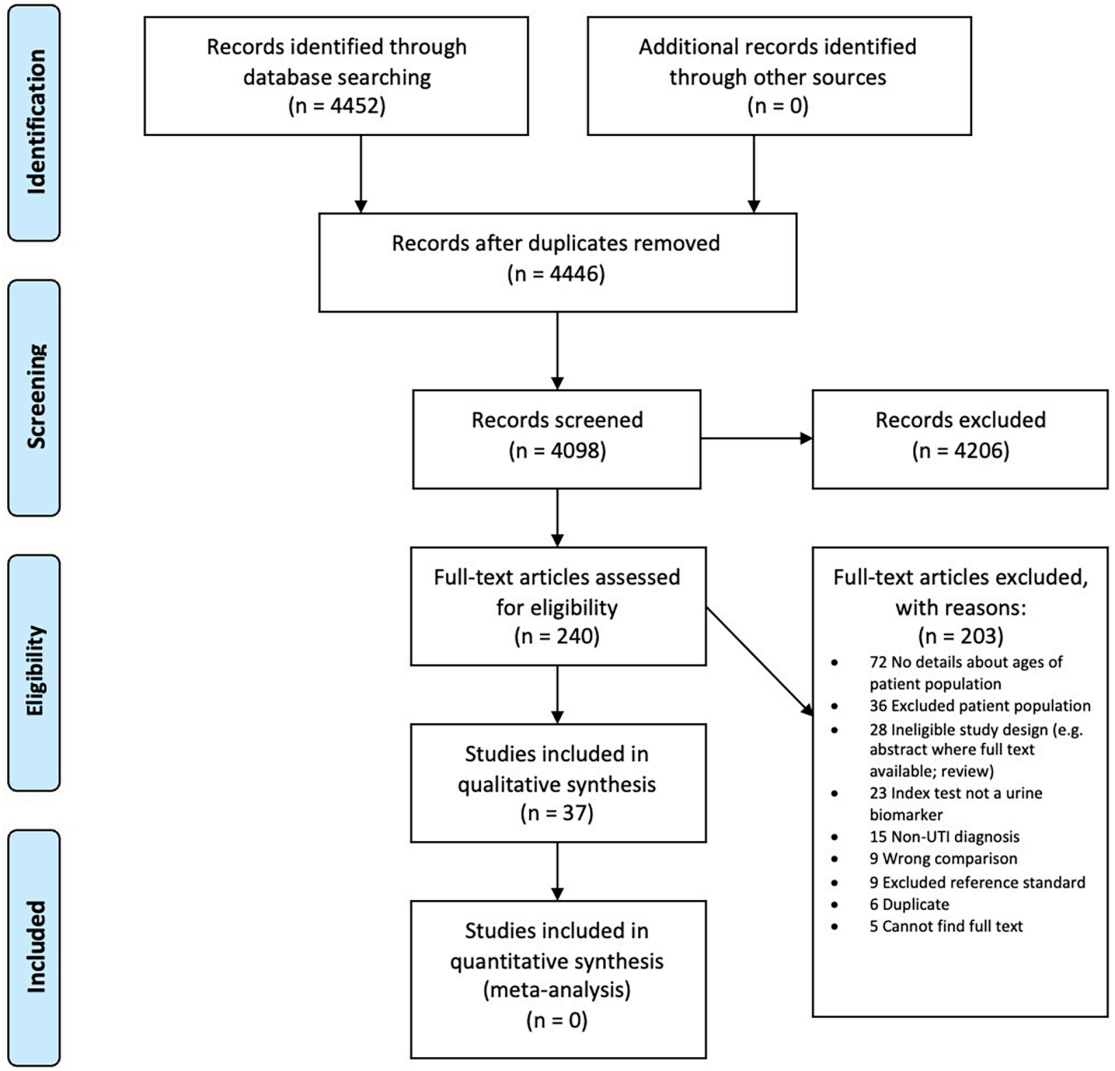
Prisma flow diagram.

Our database search identified 4446 unique references; we excluded 4206 based on the title and abstract leaving 240 for a full text review. We included 37 studies in our descriptive analysis (see Figure 1 for PRISMA flowchart). The most common reasons for exclusion were a lack of information about the ages of participants or confirmed inclusion of children. The 37 studies included 4009 adults and measured 66 different biomarkers (see Table 1 and Supplemental Table 2).

**Table 1.** Biomarker abbreviations.

Biomarker	Abbreviation
Alanine aminopeptidase	AAP
Beta-2-Microglobulin	B2M
Chemokine (C-X-C) ligand 2 (also known as monocyte chemoattractant protein 1)	CCL-2
C-X-C motif chemokine ligand 10	CXCL10
C-X-C motif chemokine ligand 5	CXCL5
Epithelial cell–derived neutrophil activating protein	ENA 78
Granulocyte colony-stimulating factor	G-CSF
C-X-C motif chemokine ligand 1	CXCL1 (GRO-a)
Heparin-Binding Protein	HBP
High Mobility Group Box-1	HMGB1
Immunoglobulin (eg, A)	Ig (eg, IgA)
Clinical Isolate Antigen	CIA
Mixed Coliform Antigen	MCA
Interleukin 1	IL-1
Interleukin 1 receptor antagonist	IL-1 RA
Interleukin 1 beta	IL-1B
Matric Metallopeptidase 9	MMP9
Urinary Neutrophil gelatinase-associated lipocalin	uNGAL
Macrophase Migration Inhibitory Factor	MIF
Myeloperoxidase to creatinine ratio	MCR
Myeloperoxidase	MPO
N-nitrosodibutylamine	NBDA
N-nitrosodiethylamine	NDEA
N-nitrosodimethylamine	NDMA
N-nitrosodi-n-propylamine	NDPA
N-nitrosodiphenylamine	NDPhA
Nerve Growth Factor	NGF
N-nitrosomethylethylamine	NMEA
N-nitrosomorpholine	NMOR
N-nitrosopiperidine	NPIP
N-nitrosopyrrolidine	NPYR
Platelet-derived growth factor	PDGF
Soluble IL-1 Receptor	sIL-1R
Soluble IL-6 Receptor	sIL-6R
Soluble tumour necrosis factor receptor (eg, 1 or 2)	sTNFR (eg, sTNFR1)
Soluble triggering receptor expressed on myeloid cells-1	sTREM-1
Trimethylamine	TMA
Tumour Necrosis Factor Alpha	TNF-alpha
Volatile Organic Compounds	VOCs

### Risk of bias assessment

Our risk of bias assessment using the QUADAS-2 tool is presented in Table 2. Overall, we found little high quality prospectively collected evidence. We found 4 studies with a prospective cohort design.^26-29^ In one of these^28^ we had low concerns in all domains apart from bias in the conduct of the index test as it wasn’t clear whether the threshold was pre-specified. This was the only study which recruited a random sample of patients presenting with a suspected UTI.^28^ We rated one of these studies with a high or medium risk of bias in all domains due to a lack of reporting of the study process.^26^

**Table 2.** Quality assessment summary using QUADAS-2 tool. Red = high risk of bias, or high concern of applicability to the research question, Orange = medium risk or concern. Green = low risk or concern.

Study	Patient selection		Index test		Reference standard		Flow and timing
	Risk of bias	Applicability	Risk of bias	Applicability	Risk of bias	Applicability	Risk of bias
*Objective 1 only*							
Bai et al^28^	Low	Low	Medium	Low	Low	Low	Low
Benlier et al^30^	High	High	High	Low	Medium	Low	High
Burdof^31^	High	High	High	Low	Low	Low	Medium
Deo and Vaidya^32^	High	Medium	High	Low	Medium	Low	Medium
Flores-Figueroa et al^27^	Medium	High	Medium	Low	Low	Low	Low
Forster et al^33^	High	High	High	Low	Medium	Low	Medium
Gadalla et al^34^	Medium	Low	Medium	Low	Low	Low	Medium
Greenwell et al^35^	High	Medium	High	Low	Low	Low	High
Hu et al^36^	High	Medium	High	Low	Low	Low	Medium
Jacobson et al^37^	High	Medium	High	Low	Low	Low	High
Johnson et al^38^	High	Medium	High	Medium	Medium	Medium	High
Kjolvmark et al^39^	High	Low	High	Low	Low	Low	High
Lam et al^40^	High	Low	High	Medium	Low	Low	Medium
Lussu et al^41^	High	Low	High	Medium	Low	Low	High
Nishitani et al^42^	High	Low	High	Low	Medium	Low	Medium
Olszyna et al^43^	High	Medium	High	Low	Medium	Medium	Medium
Olszyna et al^44^	High	Medium	High	Low	Medium	Medium	Medium
Olszyna et al^29^	Medium	Medium	High	Low	Low	Low	Medium
Price et al^22^	High	Medium	High	Low	Low	Low	Medium
Pupek-Musialik^45^	High	Low	High	Low	Low	Low	Medium
Sahin et al.^46^	Medium	Medium	High	Low	Low	Low	High
Sandberg et al^47^	High	Medium	High	Low	Low	Low	Medium
Short et al^48^	High	High	High	Low	Low	Low	High
Tyagi et al^49^	High	Low	High	Medium	Medium	Low	High
Vera et al^50^	High	Medium	High	Low	Medium	Medium	High
Wu et al^51^	Medium	Low	High	Low	Medium	Medium	Medium
Zhu et al^52^	High	Medium	High	Low	Medium	Medium	High
*Objectives 1 and 2*							
Ciszek et al^53^	High	High	High	Low	Low	Low	Medium
Determann et al^54^	High	Low	Medium	Low	Medium	Low	High
Ethel et al^55^	Medium	Low	High	Low	Low	Low	Medium
Hedges et al^56^	High	Medium	High	Low	Low	Medium	Low
Jacobson et al^57^	High	Medium	High	Low	Low	Low	High
Jacobson et al^58^	High	Medium	High	Low	Low	Low	High
Kjolvmark et al^59^	High	Medium	High	Low	Low	Low	High
Rodhe et al^60^	Medium	Medium	High	Low	Low	Low	Medium
Sundvall et al^61^	Medium	Medium	Low	Low	Medium	Low	Medium
*Objective 2 only*							
Sunden and Wullt^26^	High	Medium	High	Medium	Medium	Medium	High

Most of our included studies (33/37) had a case-control design. We rated most (26/33) of these studies were rated as having a high risk of bias for patient selection (26/33) and conduct of the index test (31/33) because they either did not use consecutive or random sampling or did not report their sampling method, and the index test was interpreted with knowledge of the results of the reference standard (culture).

Across all studies, we rated the risk of bias due to the conduct or applicability of the reference standard to be low or medium as we excluded studies not using culture and microscopy as a reference standard. We had minimal concern about the applicability of the index tests to our question, which was intentionally broad.

### Objective 1: Potential urine biomarkers for the diagnosis of UTI

The diagnostic accuracy of 64 biomarkers compared with urine culture was investigated in 36 studies including 3979 participants. Three biomarkers (MCR, IL-8, IL-6) were evaluated as part of a cohort study, and a further 7 biomarkers were each studied in 3 or more case-control studies (see Tables 3 and 4); the results for these biomarkers are summarised below. Supplemental Tables 2 and 3 detail our findings for the remaining 54 biomarkers which were studied either once or twice in 27 case-control studies. Supplemental Table 4 summarises the biological function of each biomarker.

**Table 3.** Results for IL-6 and IL-8.

Biomarker	Studies	Population	Measured biomarker (median pg/ml and IQR unless stated)								Statistical significance between UTI and non-UTI/control^a^
			UTI	Cystitis	Pyelonephritis	Febrile UTI	ASB	OAB	Not UTI	Controls	
IL-6	Ciszek et al^53^	Patients with stable graft function 12-60 month after kidney transplantation	15.71 (range (3.61-246.95)				3.92 (0.22-17.33)			3.54 (0.34-78.41)	Median IL-6 pg/mg creatinine higher (*P* = .0003) in UTI compared to ASB and control. No difference between ASB and control.
IL-6	Jacobson et al^37^	Patients admitted to hospital with non-obstructive pyelonephritis			44 (no IQR given)					nd	IL-6 was higher in patients with acute pyelonephritis than controls (*P* < .001)
IL-6	Kjolvmark et al (GP Cohort)^39^	Patients presenting in primary care with symptoms of UTI, patients presenting to ED with suspected UTI		17 (2-198)	261 (170-606)				1 (1-1)		IL-6 higher in patients with cystitis, and with pyelonephritis than patients without UTI (*P* < .01)
IL-6	Kjolvmark et al (ED Cohort)^39^	Patients presenting in primary care with symptoms of UTI, patients presenting to ED with suspected UTI		1 (1-359)	217 (7-719)				1 (1-10)		IL-6 higher in patients with pyelonephritis (*P* < .01) but not cystitis than control patients
IL-6	Kjolvmark et al^59^	Nursing home residents	150 (4-630)				4 (4-13)		4 (4-8)		Higher in UTI than ASB (*P* < .01), no difference between ASB and no UTI
IL-6	Olszyna et al^29^	165 Catheterised patients undergoing major abdominal surgery	no data							no data	IL-6 levels increased in the 2-4 days preceding UTI and a similar increase was found in patients not developing a UTI in the same period.
IL-6	Jacobson et al^57^	Patients admitted to hospital with pyelonephritis		ND (range ND-90)	44 (range ND-430)		ND (ND-24)			ND	IL-6 higher in patients with pyelonephritis than healthy controls (*P* < .001). No comparison with ASB.
IL-6	Wu et al^51^	Adult patients presenting to a hospital with UTI	5.72 (3.84-9.02)						5.31 (4.32-6.39)	5.06 (4.56-6.18)	No differences between groups were found.
IL-6	Rodhe et al^60^	Patients aged 80 and above		54.7 (range 10.7-443)			14.4 (7.1-37.4)			11.7 (5.6-69.1)	Statistical testing was performed but it was unclear which groups were compared.
IL-6	Hedges et al^56^	Non-pregnant women			81,2 units/ml (range 0-512)		86.2 units/ml (range 0/190)			Undetectable	No statistical testing reported
IL-6	Sunden et al^26^	Patients aged 80 and above	mean 227 ng/L (range 17-1400)				mean 30 ng/l (range 8-86)				Patients with sterile urine or ASB had very low or undetectable levels of urine IL-6. Patients with minor symptoms and not receiving antibiotics had lower levels of IL-6 than patients receiving antibiotics for a suspected UTI (*P* < .001). IL-6 was available to prescribing clinicians.
IL-6	Sundvall et al^61^	Nursing home residents	2.5 ng/: (1.0-5.7)						1.3 ng/L (0.6-2.8		Urine IL-6 concentration was higher in residents with positive urine culture (*P* = .000004) than in residents with negative urine culture
IL-8	Ciszek et al^53^	Patients with stable graft function 12-60 months after kidney transplantation	146.80 (range 24.65-2114.25)				33.49 (2.97-129.75)			2.97 (2.97-44.16)	Median IL-8 pg/mg creatinine higher (*P* < .001) in UTI in ASB control. IL-8 higher in ASB than control (*P* < .0004)
IL-8	Jacobson et al^57^	Patients admitted to hospital with pyelonephritis		70 (ND-840)	78 (ND-3700)		93 (ND-270)			87 (ND-720)	IL-8 higher in patients with pyelonephritis than controls (*P* < .005). No comparison made with cystitis or ASB.
IL-8	Flores-Figueroa et al^27^	Catheterised hospital patients	309						10		At a threshold of 50 pg/ml, IL-8 was associated with a sensitivity of 97% and a specificity of 85% for diagnosing UTI
IL-8	Gadalla et al^34^	Women presenting with UTI symptoms to primary care	8.57 (1.74-24.25)							4 (1-14.92)	No statistically testing performed
IL-8	Jacobson et al^37^	Patients admitted to hospital with non-obstructive pyelonephritis			870 (no IQR given)					87 (no IQR given)	IL-8 was higher in patients with acute pyelonephritis than controls (*P* < .001)
IL-8	Olszyna et al^44^	Patients with suspected gram-negative urosepsis				0.38 ng/ml (range <0.01-3.13)				<0.01 ng/ml (<0.01-0.14)	Patients with urosepsis at admission had higher urinary IL-8 than healthy controls <0.01
IL-8	Olszyna et al^29^	165 Catheterised patients undergoing major abdominal surgery	no data							no data	IL-8 was increased in UTI positive patients on the day of positive culture, but not in UTI controls (*P* < .01).
IL-8	Rodhe et al^60^	Patients aged 80 and above		313 (range 137-550)			69.6 (6.5-809)			14.5 (2.5-50.7)	Statistical difference between groups was unclear.
IL-8	Sunden et al^26^	Patients aged 80 and above	mean 2013 ng/L (range 387-3399)				mean 3392 (164-7500)				There was no different in urinary IL-8 levels between Nursing Home residents with UTI and ASB
IL-8	Tyagi et al^49^	Patients routinely visiting a urology clinic	63.67 (8.8-140.3)					4.70 (0-16.81)		4.45 (0-19.52)	UTI was higher than controls (*P* < .01), and OAB (*P* < .001)

a Statistical significance included where reported by the authors.

**Table 4.** Biomarker table for biomarkers to diagnose UTI investigated 3 times or more.

Biomarker	Studies	Population	Measured biomarker (median pg/ml and IQR unless stated)								Statistical significance between UTI and non-UTI/control^a^
			UTI	Cystitis	recurrent cystitis	Pyelonephritis	Febrile UTI	ASB	OAB	Controls	
GRO-a (CXCL1)	Rodhe et al^60^	Patients aged 80 and above		222 (range 0->6000)				38.0 (0-783)		14.5 (2.5-50.7)	Statistical testing was performed but it was unclear which groups were compared
GRO-a (CXCL1)	Olszyna et al^44^	Patients with suspected gram-negative urosepsis					0.38 ng/ml (range <0.01-3.13)			<0.01 (<0.01-0.14)	Higher in patients with febrile UTI than controls (*P* < .05)
GRO-a (CXCL1)	Tyagi et al^49^	Patients routinely visiting a urology clinic	47.14 (2.8-336.4)						4.70 (0-16.81)	4.48 (0-19.52)	Higher in samples from patients with UTI than controls and OAB (*P* < .001)
IgA – secretory	Ethel et al^55^*	Women (no more details given)	mean 0.045 (±0.04) urinary antibodies to mixed coliform antigen					mean 0.101 (±0.13) urinary antibodies to mixed coliform antigen		mean 0.44 (±0.02) urinary antibodies to mixed coliform antigen	Higher in samples from patients with ASB (*P* < .01)
IgA – secretory	Deo and Vaidya^32^	Adult patients showing signs of urinary tract infection	mean 80 ug/ml (±48)							mean 5.2 (±0.73)	Higher in samples from patients with UTI than those from controls (*P* < .001)
IgA – secretory	Greenwell et al^35^	Adults (no more details given)		1		1				1	No difference between samples from patients with cystitis and controls. Higher in samples from patients with pyelonephritis than controls (*P* = .012)
IgA – secretory	Short et al^48^	Catheterised women with recurrent cystitis			mean 0.774 mg/dl creatinine (±0.186)					mean 0.228 mg/dl creatinine (±0.140)	Higher in samples from patient with UTI than controls (*P* < .001)
IL-1 RA	Jacobson et al^58^	Patients admitted to hospital with non-obstructive pyelonephritis				40 (range nd-43 000)		nd (nd-52)		8400 (700-76 000)	Lower in patients with pyelonephritis than controls (*P* < .001). No comparison with ASB
IL-1 RA	Jacobson et al^37^	Patients admitted to hospital with non-obstructive pyelonephritis				40 (no IQR given)				8400 (no IQR given)	Lower in patients with pyelonephritis than controls (*P* < .001).
IL-1 RA	Olszyna et al^43^	No detail given					0.67 ng/ml (range 0.10-26.0)			0.60 (<0.08-3.62)	No differences found
IL-1 RA	Tyagi et al^49^	Patients routinely visiting a urology clinic	72.08 (28.45-151.7)						62.03 (33.07-148.1)	181.9 (40.02-635.6)	Higher in samples from patients with UTI than controls and OAB (*P* < .05)
IL-10	Rodhe et al^60^	Patients aged 80 and above		12.5 (1.4-31.3)				14.6 (5.3-31.6)		9.2 (1.7-16.6)	Statistical testing was performed but it was unclear which groups were compared
IL-10	Jacobson et al^37^	Patients admitted to hospital with non-obstructive pyelonephritis				nd				nd	No differences found
IL-10	Olszyna et al^43^	No detail given					nd			nd	No differences found
IL-1B	Rodhe et al^60^	Patients aged 80 and above		97.1 (0-11 500)				62.7 (15.9-153)		57.5 (25.9-186)	Statistical testing was performed but it was unclear which groups were compared
IL-1B	Olszyna et al^43^	No detail given					nd			nd	No differences found
IL-1B	Tyagi et al^49^	Patients routinely visiting a urology clinic	1.76 (0.84-5.26)						0.12 (0.07-0.23)	0.29 (0.06-2.24)	No differences found
sTNFR1	Jacobson et al^58^	Patients admitted to hospital with non-obstructive pyelonephritis				2500 (range 334-24 000)		460 (206-1040)		1125 (195-3250)	Higher in patients with pyelonephritis than controls (*P* < .001). No comparison with ASB.
sTNFR1	Jacobson et al^37^	Patients admitted to hospital with non-obstructive pyelonephritis				2500 (no IQR given)				1125 (no IQR given)	Higher in patients with pyelonephritis than controls (*P* < .001).
sTNFR1	Olszyna et al^43^	No detail given					17.55 ng/ml (range <0.04-25.0)			1.78 (0.4-3.32)	Higher in patients with febrile UTI than controls (*P* < .001)
sTNFR2	Jacobson et al^58^	Patients admitted to hospital with non-obstructive pyelonephritis				6300 (940-54 000)		980 (424-3000)		1980 (375-5000)	Higher in patients with pyelonephritis than controls (*P* < .001). No comparison with ASB.
sTNFR2	Jacobson et al^37^	Patients admitted to hospital with non-obstructive pyelonephritis				6300 (no IQR given)				1980 (no IQR given)	Higher in patients with pyelonephritis than controls (*P* < .001).
sTNFR2	Olszyna et al^43^	No detail given					14.96 ng/ml (range <0.02-50.0)			2.34 (0.5-5.34)	Higher in patients with febrile UTI than controls (*P* < .001)

a Statistical significance included where reported by the authors.

### Myeloperoxidase (MPO) to creatinine ratio (MCR)

One cohort study measured myeloperoxidase (MPO) to creatinine ratio (MCR) (measured in ng MPO to g creatinine) in 253 adult outpatients with suspected UTI.^28^ In samples which were culture positive for 1 or 2 pathogens, log_2_MCR values were higher than those in patients with sterile urine (mean 8.6 ng/g (SD 2.5) vs 5.4 (SD 1.5), *P* = .001). Accordingly an MCR of 194.0 ng/g or above had a sensitivity of 66%, specificity of 95% and PPV of 95% for positive culture.

### IL-8

**Figure 2.**
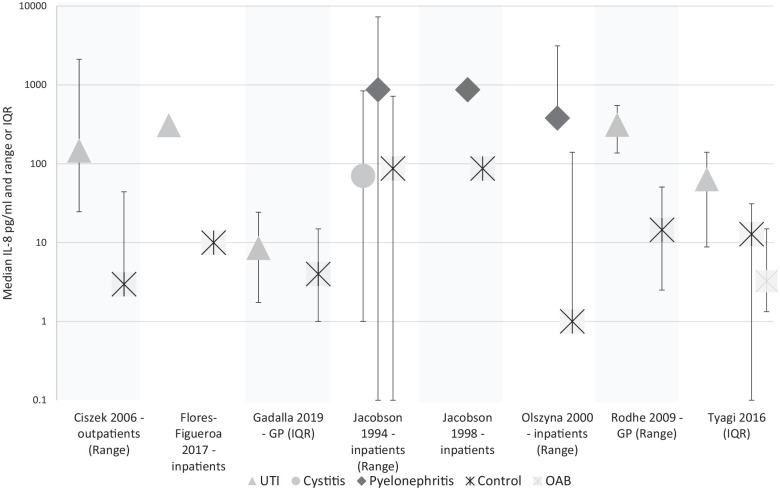
Median IL-8 titre in pg/ml in studies reporting medians. Olszyna et al.^29^ not shown. Variance shown in the study title.

We found 2 cohort studies of catheterised in-patients and 7 case-control studies, conducted in hospital in-patients, out-patients and general practice. Figure 2 displays median IL-8 levels measured for each condition across these studies.

In a cohort study of 16 catheterised in-patients,^27^ a threshold of 50pg IL-8 per ml of urine was associated with a sensitivity of 97.1% (95% CI 77.1-99.9) and a specificity of 85.3% (95% CI 62.3-95.3) for diagnosing UTI.

In a second cohort study of 165 patients catheterised following major abdominal surgery,^29^ mean IL-8 concentrations were higher at the point of positive urine culture than baseline, and also higher than in patients without a positive culture. No precise figures were given.

The results from the case-control studies are summarised in Table 3. Urinary IL-8 was between 10 and 36 times higher in samples from patients with pyelonephritis^37,57^ and febrile UTI,^44^ and between 2 and 49.4 times higher in samples from patients with cystitis or a non-specific UTI (ie, a UTI not specified as upper or lower tract) than in those from healthy controls.^34,49,53,60^ One study, including 8 participants with cystitis found similar median urinary IL-8 in cystitis and control groups.^57^

### IL-6

**Figure 3.**
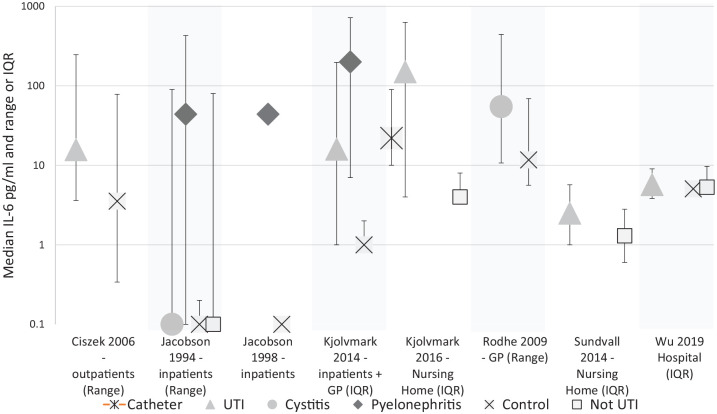
Median IL-6 titre in pg/ml in studies reporting medians. Olszyna et al.^29^ and Hedges et al.^56^ not shown. Variance shown in study title.

We found 1 cohort study, 1 cross-sectional study and 7 case-control studies investigating IL-6, conducted across in-patient, nursing home and primary care populations. See Figure 3 for median IL-6 levels reported in these studies.

In the cohort study, no increase in mean IL-6 the urine of catheterised patients on the day of UTI diagnosis was detected. This study found that urinary IL-6 increased in all patients in the 8 days following catheterisation.^29^

In the cross-sectional study, urinary IL-6 was found to be higher in nursing home residents with positive urine culture (median concentration of 2.5 ng/l (range 1.0-5.7)) compared with those with negative urine culture 1.3 ng/l (range 0.6-2.8) *P* < .001.

The results from the case-control studies are summarised in Table 3. Three studies found higher level of IL-6 in samples from patients with pyelonephritis than samples from healthy control subjects.^37,39,57^ Six studies compared samples from patients with cystitis or a non-specific UTI with those from controls.^39,51,53,57,59,60^ In 3 studies, samples from patients were between 3.8 and 37.5 times higher in samples from patients than those from controls.^53,59,60^ The remaining 3 found equivocal results,^39^ or no difference between groups.^51,57^

#### Other biomarkers

##### We found consistent evidence that biomarkers CXCL-1 and sTNFR types 1 and 2 were elevated in samples from patients with UTI

Three studies found median chemokine (C-X-C Motif) ligand 1 (CXCL-1) to be between 10 and 38 times higher in samples from a mixed population of patients with UTI, cystitis, febrile UTI compared with controls.^44,49,60^ Median levels of sTNFR-1 were between 2 and 10 times higher in samples from patients with pyelonephritis or febrile UTI than in those from healthy controls, whilst median sTNRF-2 was between 3 and 7 times higher.^37,43,58^

##### We found consistent evidence that IL-1B and IL-10 biomarkers were not elevated in samples from patients with UTI

We found 3 studies each which demonstrated no elevation of IL-1B^43,49,60^ or IL-10 marker levels in samples from patients with UTI.^37,43,60^

##### We found contradictory evidence for 2 biomarkers: secretory IgA (sIgA) and IL1-RA

Two studies found samples from patients with recurrent UTI or UTI not specified as upper or lower tract to have mean levels of sIgA between 3.3 and 40 times higher than healthy controls.^32,48^ Two studies found no difference in mean concentration of urinary sIgA between cystitis and non-specific UTI groups, and controls respectively.^35,55^

Two studies from the same trial found median IL1-RA concentrations to be 200 times lower (*P* < .001) in patients with UTI than controls, albeit with large ranges. One study found median IL1-RA in samples from patients with UTI to be half (*P* < .05) that of samples from healthy controls.^49^ One study found no differences.^43^

### Objective 2: Biomarkers to distinguish between asymptomatic bacteriuria and UTI

We found 9 studies, including 1058 patients, and evaluating 14 biomarkers for differentiating urine from patients with positive culture and symptoms (UTI) and positive culture and no symptoms (asymptomatic bacteriuria (ASB)) (Tables 3 and 4). One study had a cohort design, whilst the rest had a case-control design. Seven studies compared ASB to an non-specific UTI group^26,53-55,57,60,61^, 1 study compared ASB to a cystitis and a pyelonephritis group,^59^ and the final study compared ASB with a pyelonephritis group only.^56^ Fourteen biomarkers were investigated; IL-6 7 times, IL-8 4 times, and the remainder only once.

All of these studies compared biomarker levels in patients with a positive urine culture and symptoms to patients who had a positive urine culture without symptoms. No study attempted to differentiate UTI and ASB in patients who were unable to report symptoms. In 1 study,^26^ biomarkers levels were compared between patients with UTI symptoms prescribed antibiotics and those not receiving antibiotics, although the biomarker was made available to clinicians making treatment decisions, meaning there may be an incorporation bias.

### IL-8

Four studies analysed IL-8, one of which had a cohort design and sampled a nursing home population.^26^ The cohort study took monthly urine samples from 35 patients with ASB (defined as 2 consecutive urine samples 4 weeks apart with ⩾10^5^ CFU/ml of the same pathogen with controlled somatic diseases) and compared levels of both biomarkers before and during episodes of UTI. During ASB, the mean IL-8 concentration in urine was 2013 ng/l (range 387-3999) in comparison to 3392 (164-7500) during UTI. No statistical difference was found.

In one case-control study urine samples from patients with cystitis^60^ had a median concentration of IL-8 4.8 times higher than those from patients with ASB. One study including renal transplant patients with non-specific UTI during follow up demonstrated that those with UTI had a median concentration of IL-8 4.4 times higher than samples from patients with ASB (*P* < .001).^53^ A study including patients with pyelonephritis and cystitis found that they had comparable Il-8 concentrations to patients with ASB.^57^

### IL-6

Six studies analysed IL-6, including one cohort study.^26^ In the cohort study, the mean IL-6 concentration in urine from patients with ASB was 30 ng/l (range 8-86) in comparison to a mean of 227 (17-1400) during UTI (*P* = .017).

In a second phase of the cohort study, 254 suspected UTIs in 84 patients were analysed using IL-6 testing in combination with a urine culture. Patients with minor symptoms and not receiving antibiotics had lower levels of IL-6 than patients receiving antibiotics for a suspected UTI (point estimates not given) (*P* < .0001).^26^ At a threshold of 25 ng/ml, urinary IL-6 was associated with a sensitivity of 57%, a specificity of 80%, a PPV of 52% and an NPV of 83% for differentiating treated UTI from ASB.

Two case-control studies found 4^53^ and 37.5^59^ times higher median urinary IL-6 in samples from patients with non-specific UTI than those with ASB (see Table 3). One study found median IL-6 at 3.8 times the concentration in samples from patients with cystitis compared to those from patients with ASB^60^, whilst another found no difference.^57^ Two studies found no differences in urinary IL-6 between samples from patients with pyelonephritis and those with ASB.^56,57^

### Others

Results from the remaining 12 biomarkers are summarised in Supplemental Table 2. Three biomarkers: uHPB, CXCL-1 and sTREM-1 were elevated in samples from patients with UTI compared to those with ASB. In the study analysing sTREM-1, 39/70 participants with a UTI (56%) had a urine culture, whilst the remainder we diagnosed with a positive dipstick and bacteria in the urine sediment.

## Discussion

We found 37 studies investigating 64 individual urine biomarkers for the diagnosis of UTI. The quality of the available evidence was limited by study design and heterogeneity, but a handful of biomarkers emerged as viable candidates for new diagnostic tests. In 8 studies IL-8 was consistently raised in UTI patients compared to controls, and in 1 study rose 24 hours earlier than a UTI diagnosis was made. CXCL-1 was also markedly higher in samples from patients with UTI compared to those from controls across 3 studies, albeit all were case-control. A singular cohort study suggested MCR may have a good ability to rule-in UTI, but the sensitivity was low. In the majority of studies, IL-6 was also associated with infection compared to controls, and was the only biomarker to consistently demonstrate higher levels in UTI compared to ASB, including in older adults.

The role of specific cytokines in the bladder’s response to infection is a major unknown. Interestingly IL-8, IL-6 and CXCL-1 are released by macrophages, the largest resident immune cell population in the bladder, and the urothelium.^62^ Early in pathogenic *E. coli* infection, shedding of superficial urothelial cells helps clear bacteria, but also exposes deeper layers of the urothelium, triggering cytokine release. A delicate balance exists between bacterial clearance and preserving tissue integrity. Taken together, high levels of these cytokines may be early indicators of invasive infection and help differentiate this from asymptomatic bacteruria.

Our results incorporate a major expanse in evidence since a previous systematic review in 2009,^18^ which only identified 11 urine biomarkers. The previous review also focused on serum biomarkers, including IL-6, IL-8 and procalcitonin, which cannot differentiate UTI from other sources of infection. The evidence for urinary IL-6 and IL-8 is mirrored in a recent systematic review of febrile children with UTI; urinary IL-6 had a pooled sensitivity of 77% (95% CI 69%-83%) and specificity of 87% (95% CI 86%-92%), and urinary IL-8 had a pooled sensitivity of 87% (95% CI 82%-91%) and specificity of 90% (95% CI 87%-93%).^63^

Due to the nature and quality of evidence available there are some limitations to our findings. There was virtually no data on diagnostic performance, hence we were unable to perform quantitative analysis, either between biomarkers, or compared to dipstick or culture. The majority of biomarkers were only studied once and the large heterogeneity between study design, population ages and included conditions make comparisons less meaningful. We did not use a pre-specified threshold for number of colony forming units per ml of urine in our reference standard. This reflects the lack of consensus on this threshold internationally.^21,22^ We also included microscopy as a possible, albeit far from perfect, reference standard, and found one study which used microscopy only as the reference standard for some patients. The diagnostic accuracy of microscopy is unclear due to a lack of specific evidence and its sensitivity may be as low as 50%,^64^ however it may be used to rule out the presence of bacteria. Both choices allowed us to capture the widest range of evidence possible regarding novel biomarkers, but may have reduced comparability of evaluations. Furthermore, most of our included studies had a case-control design increasing the risk of selection and ascertainment bias. All of the studies comparing biomarkers in symptomatic UTI versus ASB used patient-reported conventional UTI symptoms to distinguish between the 2 conditions. This limits the applicability to patients with atypical symptoms, or those who cannot communicate, such as nursing home residents.

Our results are a stepping-stone for future research, in particular prospective, rather than case-control studies. Larger cohort studies could help determine diagnostic performance and thresholds for testing. Our focus would be on settings where near-patient testing has the potential for largest impact, for example, within primary care, where urine dipsticks are usually the only other test available, and delays in receiving urine culture results are longest. Research addressed at how novel biomarkers may differentiate between ASB and UTI need careful design. Currently there is no universal reference standard, and there is also an overlap in patient populations, especially in older adults, as patients with ASB develop active infection. Combined panels of urine biomarkers may also increase diagnostic performance. In one study an algorithm using IL-8 and 3 other biomarkers (MMP9, NGAL and IL-1β) had a modest ability to rule in infections (positive Likelihood ratio 6.29, 95% CI 2.04-19.36),^34^ although the diagnostic thresholds used in these algorithms were not specified. Future research may consider the diagnostic value of measuring multiple biomarkers from a single sample, and the added value of this for clinicians. Ultimately, well powered and carefully design randomised trials of urinary biomarkers in practice are needed to establish how they can help identify those patients who would most benefit from antibiotics.

## Conclusion

This systematic review provides justification for the further investigation of a number of novel urinary biomarkers, notably CXCL-1, IL-6, IL-8, MCR and the sTNFRs. Primary care based prospective studies are needed to establish diagnostic performance and utility in clinical practice.

## Supplemental Material

sj-docx-1-bmi-10.1177_11772719221144459 – Supplemental material for What is the Diagnostic Accuracy of Novel Urine Biomarkers for Urinary Tract Infection? Click here for additional data file. Supplemental material, sj-docx-1-bmi-10.1177_11772719221144459 for What is the Diagnostic Accuracy of Novel Urine Biomarkers for Urinary Tract Infection? by George Edwards, Anna Seeley, Adam Carter, Maia Patrick Smith, Elizabeth LA Cross, Kathryn Hughes, Ann Van den Bruel, Martin J Llewelyn, Jan Y Verbakel and Gail Hayward in Biomarker Insights

sj-docx-4-bmi-10.1177_11772719221144459 – Supplemental material for What is the Diagnostic Accuracy of Novel Urine Biomarkers for Urinary Tract Infection? Click here for additional data file. Supplemental material, sj-docx-4-bmi-10.1177_11772719221144459 for What is the Diagnostic Accuracy of Novel Urine Biomarkers for Urinary Tract Infection? by George Edwards, Anna Seeley, Adam Carter, Maia Patrick Smith, Elizabeth LA Cross, Kathryn Hughes, Ann Van den Bruel, Martin J Llewelyn, Jan Y Verbakel and Gail Hayward in Biomarker Insights

sj-docx-6-bmi-10.1177_11772719221144459 – Supplemental material for What is the Diagnostic Accuracy of Novel Urine Biomarkers for Urinary Tract Infection? Click here for additional data file. Supplemental material, sj-docx-6-bmi-10.1177_11772719221144459 for What is the Diagnostic Accuracy of Novel Urine Biomarkers for Urinary Tract Infection? by George Edwards, Anna Seeley, Adam Carter, Maia Patrick Smith, Elizabeth LA Cross, Kathryn Hughes, Ann Van den Bruel, Martin J Llewelyn, Jan Y Verbakel and Gail Hayward in Biomarker Insights

sj-xlsx-1-bmi-10.1177_11772719221144459 – Supplemental material for What is the Diagnostic Accuracy of Novel Urine Biomarkers for Urinary Tract Infection? Click here for additional data file. Supplemental material, sj-xlsx-1-bmi-10.1177_11772719221144459 for What is the Diagnostic Accuracy of Novel Urine Biomarkers for Urinary Tract Infection? by George Edwards, Anna Seeley, Adam Carter, Maia Patrick Smith, Elizabeth LA Cross, Kathryn Hughes, Ann Van den Bruel, Martin J Llewelyn, Jan Y Verbakel and Gail Hayward in Biomarker Insights

sj-xlsx-3-bmi-10.1177_11772719221144459 – Supplemental material for What is the Diagnostic Accuracy of Novel Urine Biomarkers for Urinary Tract Infection? Click here for additional data file. Supplemental material, sj-xlsx-3-bmi-10.1177_11772719221144459 for What is the Diagnostic Accuracy of Novel Urine Biomarkers for Urinary Tract Infection? by George Edwards, Anna Seeley, Adam Carter, Maia Patrick Smith, Elizabeth LA Cross, Kathryn Hughes, Ann Van den Bruel, Martin J Llewelyn, Jan Y Verbakel and Gail Hayward in Biomarker Insights

sj-xlsx-5-bmi-10.1177_11772719221144459 – Supplemental material for What is the Diagnostic Accuracy of Novel Urine Biomarkers for Urinary Tract Infection? Click here for additional data file. Supplemental material, sj-xlsx-5-bmi-10.1177_11772719221144459 for What is the Diagnostic Accuracy of Novel Urine Biomarkers for Urinary Tract Infection? by George Edwards, Anna Seeley, Adam Carter, Maia Patrick Smith, Elizabeth LA Cross, Kathryn Hughes, Ann Van den Bruel, Martin J Llewelyn, Jan Y Verbakel and Gail Hayward in Biomarker Insights

## Declarations

**Ethics approval and consent to participate:** Not required.

**Consent for publication:** All authors have approved the manuscript and consented to its publication.

**Availability of data and materials:** Not applicable.

## References

[1] Medina-Bombardó D Jover-Palmer A . Does clinical examination aid in the diagnosis of urinary tract infections in women? A systematic review and meta-analysis. BMC Fam Pract. 2011;12:111. 21985418 10.1186/1471-2296-12-111 PMC3207883

[2] Foxman B . The epidemiology of urinary tract infection. Nat Rev Urol. 2010;7:653-660. 21139641 10.1038/nrurol.2010.190

[3] Dolk FCK Pouwels KB Smith DRM Robotham JV Smieszek T . Antibiotics in primary care in England: which antibiotics are prescribed and for which conditions? J Antimicrob Chemother. 2018;73:ii2-ii10. 10.1093/jac/dkx504 PMC5890730 29490062

[4] Gbinigie OA Onakpoya IJ Richards GC , et al. Biomarkers for diagnosing serious bacterial infections in older outpatients: a systematic review. BMC Geriatr. 2019;19:1-9. 31315578 10.1186/s12877-019-1205-0 PMC6637629

[5] Nicolle LE Gupta K Bradley SF , et al. Clinical practice guideline for the management of asymptomatic bacteriuria: 2019 update by the infectious diseases society of America. Clin Infect Dis. 2019;68:E83-E75. 10.1093/cid/ciy1121 30895288

[6] Chin TL McNulty C Beck C MacGowan A . Antimicrobial resistance surveillance in urinary tract infections in primary care. J Antimicrob Chemother. 2016;71:2723-2728. 27353470 10.1093/jac/dkw223

[7] Costelloe C Metcalfe C Lovering A Mant D Hay AD . Effect of antibiotic prescribing in primary care on antimicrobial resistance in individual patients: systematic review and meta-analysis. Br Med J. 2010;340:1120. 10.1136/bmj.c2096 20483949

[8] Cai T Mazzoli S Mondaini N , et al. The role of asymptomatic bacteriuria in young women with recurrent urinary tract infections: to treat or not to treat? Clin Infect Dis. 2012;55:771-777. 22677710 10.1093/cid/cis534

[9] NICE. Urinary tract infection (recurrent): antimicrobial prescribing. NICE guideline. Ng112. 2018;(October):1-34.

[10] NICE. Urinary tract infection (lower) - women. 2021. https://cks.nice.org.uk/topics/urinary-tract-infection-lower-women/

[11] Woodford HJ George J . Diagnosis and management of urinary tract infection in hospitalized older people. J Am Geriatr Soc. 2009;57:107-114. 19054190 10.1111/j.1532-5415.2008.02073.x

[12] Giesen LG Cousins G Dimitrov BD van de Laar FA Fahey T . Predicting acute uncomplicated urinary tract infection in women: a systematic review of the diagnostic accuracy of symptoms and signs. BMC Fam Pract. 2010;11:78. 20969801 10.1186/1471-2296-11-78 PMC2987910

[13] Devillé WL Yzermans JC van Duijn NP Bezemer PD van der Windt DA Bouter LM . The urine dipstick test useful to rule out infections. A meta-analysis of the accuracy. BMC Urol. 2004;4:14. 15175113 10.1186/1471-2490-4-4 PMC434513

[14] Ducharme J Neilson S Ginn JL . Can urine cultures and reagent test strips be used to diagnose urinary tract infection in elderly emergency department patients without focal urinary symptoms? Can J Emerg Med. 2007;9:87-92. 10.1017/s1481803500014846 17391578

[15] Spek M Cals JWL Oudhuis GJ Savelkoul PHM de Bont EGPM . Workload, diagnostic work-up and treatment of urinary tract infections in adults during out-of-hours primary care: a retrospective cohort study. BMC Fam Pract. 2020;21:1-7. 33172396 10.1186/s12875-020-01305-8 PMC7653778

[16] Ordóñez-Mena JM Fanshawe TR Foster D , et al. Frequencies and patterns of microbiology test requests from primary care in Oxfordshire, UK, 2008-2018: a retrospective cohort study of electronic health records to inform point-of-care testing. BMJ Open. 2021;11:1-9. 10.1136/bmjopen-2020-048527 PMC8611454 34815274

[17] Nicolle LE . Asymptomatic bacteriuria in the elderly. Infect Dis Clin North Am. 1997;11:647-662. 9378928 10.1016/s0891-5520(05)70378-0

[18] Nanda N Juthani-Mehta M . Novel biomarkers for the diagnosis of urinary tract infection-a systematic review. Biomark Insights. 2009;4:S3155. 10.4137/bmi.s3155 PMC2729697 19707519

[19] Strimbu K Tavel JA . What are biomarkers? Curr Opin HIV AIDS. 2010;5:463-466. 20978388 10.1097/COH.0b013e32833ed177 PMC3078627

[20] Boon HA Struyf T Bullens D Van den Bruel A Verbakel JY . Diagnostic value of biomarkers for paediatric urinary tract infections in primary care: systematic review and meta-analysis. BMC Fam Pract. 2021;22:1-12. 34565335 10.1186/s12875-021-01530-9 PMC8474745

[21] Stamm WE Counts GW Running KR Fihn S Turck M Holmes KK . Diagnosis of coliform infection in acutely dysuric women. N Engl J Med. 1982;307:463-468. 7099208 10.1056/NEJM198208193070802

[22] Price TK Dune T Hilt EE , et al. The clinical urine culture: enhanced techniques improve detection of clinically relevant microorganisms. J Clin Microbiol. 2016;54:1216-1222. 26962083 10.1128/JCM.00044-16 PMC4844725

[23] Wilson ML Gaido L . Laboratory diagnosis of urinary tract infections in adult patients. Clin Infect Dis. 2004;38:1150-1158. 15095222 10.1086/383029

[24] Covidence. 2022 Covidence. https://www.covidence.org/

[25] Whiting PF Reitsma JB Leeflang MMG , et al. Research and reporting methods accuracy studies. Ann Intern Med. 2011;155:529-536. 22007046 10.7326/0003-4819-155-8-201110180-00009

[26] Sundén F Wullt B . Predictive value of urinary interleukin-6 for symptomatic urinary tract infections in a nursing home population. Int J Urol. 2016;23:168-174. 26552369 10.1111/iju.13002

[27] Flores-Figueroa J Ortiz-Navarrete V Paredes-Paredes M Espinosa-Lopez FR Castro-D'Franchis LJ . Urinary IL-8 as an early diagnostic tool for nosocomial urinary tract infection. Med Sci Technol. 2017;58:15-20.

[28] Bai MJ Jing F Guowei L . Urinary myeloperoxidase to creatinine ratio as a new marker for diagnosis of urinary tract infection. Chinese Med Sci J. 2018;33:152-159. 10.24920/11814 30266105

[29] Olszyna DP Vermeulen H Baan AH , et al. Urine interleukin-8 is a marker for urinary tract infection in postoperative patients. Infection. 2001;29:274-277. 11688906 10.1007/s15010-001-1157-z

[30] Benlier N Solakhan M Yıldırım Z , et al. A novel diagnostic tool for the detection of bladder cancer: measurement of urinary high mobility group box-1. Urol Oncol Semin Original Investig. 2020;38:685.e11-685.e16. 10.1016/j.urolonc.2020.03.025 32312640

[31] Burdof DW . Quantitative studies on urinary immunoglobulins in hospital patients, including patients with urinary tract infection. Clin Exp Immunol. 1970;6:189-18996. 5435715 PMC1712771

[32] Deo SS Vaidya AK . Elevated levels of secretory immunoglobulin A (sIgA) in urinary tract infections. Indian J Pediatr. 2004;71:37-40. 14979384 10.1007/BF02725654

[33] Forster CS Lamanna OK Rounds A Sprague BM Ljungberg I Groah SL . The association between urine neutrophil gelatinase-associated lipocalin and UTI in people with neurogenic lower urinary tract dysfunction. Spinal Cord. 2021;59:959-966. 32963362 10.1038/s41393-020-00552-x PMC11702270

[34] Gadalla AAH Friberg IM Kift-Morgan A , et al. Identification of clinical and urine biomarkers for uncomplicated urinary tract infection using machine learning algorithms. Sci Rep. 2019;9:1-11. 31873085 10.1038/s41598-019-55523-x PMC6928162

[35] Greenwell D Petersen J Kulvicki A Harder J Goldblum R Neal DE . Urinary secretory immunoglobulin A and free secretory component in pyelonephritis. Am J Kidney Dis. 1995;26:590-594. 7573012 10.1016/0272-6386(95)90594-4

[36] Hu CW Shih YM Liu HH Chiang YC Chen CM Chao MR . Elevated urinary levels of carcinogenic N-nitrosamines in patients with urinary tract infections measured by isotope dilution online SPE LC–MS/MS. J Hazard Mater. 2016;310:207-216. 26937867 10.1016/j.jhazmat.2016.02.048

[37] Jacobson SH Lu Y Brauner A . Soluble interleukin-6 receptor, interleukin-10 and granulocyte colony-stimulating factor in acute pyelonephritis: relationship to markers of bacterial virulence and renal function. Nephron. 1998;80:401-407. 9832638 10.1159/000045211

[38] Johnson EU Probert CS Persad R Khalid T Ratcliffe N . 676 urinary volatile organic compounds: novel approach to rapid UTI diagnosis. Eur Urol Suppl. 2014;13:e676.

[39] Kjölvmark C Påhlman LI Åkesson P Linder A . Heparin-binding protein: a diagnostic biomarker of urinary tract infection in adults. Open Forum Infect Dis. 2014;1:ofu004. 10.1093/ofid/ofu004 PMC4324176 25734078

[40] Lam CW Law CY To KKW , et al. NMR-based metabolomic urinalysis: a rapid screening test for urinary tract infection. Clin Chim Acta. 2014;436:217-223. 24909875 10.1016/j.cca.2014.05.014

[41] Lussu M Camboni T Piras C , et al. ^1^H NMR spectroscopy-based metabolomics analysis for the diagnosis of symptomatic *E. coli*-associated urinary tract infection (UTI). BMC Microbiol. 2017;17:201. 28934947 10.1186/s12866-017-1108-1 PMC5609053

[42] Nishitani Y Kubo A Kaneko Y , et al. Increased urinary levels of adrenomedullin in patients with cystitis. Am J Kidney Dis. 1999;33:772-777. 10196022 10.1016/s0272-6386(99)70232-5

[43] Olszyna DP Prins JM Buis B van Deventer SJH Speelman P van der Poll T . Levels of inhibitors of tumor necrosis factor alpha and interleukin 1β in urine and sera of patients with urosepsis. Infect Immun. 1998;66:3527-3534. 9673230 10.1128/iai.66.8.3527-3534.1998 PMC108383

[44] Olszyna D Opal S Prins J , et al. Chemotactic activity of CXC chemokines interleukin-8, growth-related oncogene–α, and epithelial cell–derived neutrophil-activating protein–78 in urine of patients with urosepsis. J Infect Dis. 2000;182:1731-1737. 11069246 10.1086/317603

[45] Pupek-Musialik D . Usefulness of beta-2-microglobulin determination in the differential diagnosis of infections in the upper and lower parts of the urinary tract. Wiad Lek. 1990;43:183-187. 2195774

[46] Şahin K Dilek AR Güvendağ Güven ES Yazıcı ZA . Contribution of neutrophil activation in the differentiation of urine infection and contamination in pregnant women. Gynecol Obstet Invest. 2015;80:124-127. 25998166 10.1159/000381898

[47] Sandberg T Bergmark J Hultberg B Jagenburg R Trollfors B . Diagnostic potential of urinary enzymes and β2-microglobulin in acute urinary tract infection. Acta Med Scand. 2009;219:489-495. 10.1111/j.0954-6820.1986.tb03344.x 2874689

[48] Short KL West CA Brinson D , et al. Comparison of 0 antigen-specific urinary immunoglobulins to *Escherichia coli* in normal women and women prone to *Escherichia coli* cystitis. Br J Urol. 1987;60:47-50. 2441789 10.1111/j.1464-410x.1987.tb09132.x

[49] Tyagi P Tyagi V Qu X , et al. Identification of clinical and urine biomarkers for uncomplicated urinary tract infection using machine learning algorithms. Sci Rep. 2019;9:1-11. 31873085 10.1038/s41598-019-55523-x PMC6928162

[50] Vera PL Preston DM Moldwin RM , et al. Elevated urine levels of macrophage migration inhibitory factor in inflammatory bladder conditions: a potential biomarker for a subgroup of interstitial cystitis/bladder pain syndrome patients. Urology. 2018;116:55-62. 29580781 10.1016/j.urology.2018.02.039 PMC5975106

[51] Wu Y Zheng W Li J Cao Y Wu Y . Diagnostic value of urine heparin-binding protein, interleukin-6 and white blood cell in bacterial urinary tract infection. Chin J Lab Med. 2019;42:312-317.

[52] Zhu X Qiao Y Liu W , et al. CXCL5 is a potential diagnostic and prognostic marker for bladder cancer patients. Tumor Biol. 2016;37:4569-4577. 10.1007/s13277-015-4275-4 26503215

[53] Ciszek M Pączek L Bartłomiejczyk I Mucha K . Urine cytokines profile in renal transplant patients with asymptomatic bacteriuria. Transplantation. 2006;81:1653-1657. 16794530 10.1097/01.tp.0000226072.20185.f8

[54] Determann RM Schultz MJ Geerlings SE . Soluble triggering receptor expressed on myeloid cells-1 is not a sufficient biological marker for infection of the urinary tract. J Infect. 2007;54:e249-e250. 10.1016/j.jinf.2007.01.010 17343917

[55] Ethel S Bhat G Hegde B . Bacterial adherence and humoral immune response in women with symptomatic and asymptomatic urinary tract infection. Indian J Med Microbiol. 2006;24:30-33. 16505552 10.4103/0255-0857.19891

[56] Hedges S Stenqvist K Lidin-Janson G Martinell J Sandberg T Svanborg C . Comparison of urine and serum concentrations of interleukin-6 in women with acute pyelonephritis or asymptomatic bacteriuria. J Infect Dis. 1992;166:653-656. 1500753 10.1093/infdis/166.3.653

[57] Jacobson SH Hylander B Wretlind B Brauner A . lnterleukin-6 and lnterleukin-8 in serum and urine in patients with acute pyelonephritis in relation to bacterial-virulence-associated traits and renal function. Nephron. 1994;67:172-179. 7915403 10.1159/000187923

[58] Jacobson SH Lu Y Brauner A . Tumour necrosis factor soluble receptors I and II and interleukin-1 receptor antagonist in acute pyelonephritis in relation to bacterial virulence-associated traits and renal function. Nephrol Dial Transplant. 1996;11:2209-2214. 8941580 10.1093/oxfordjournals.ndt.a027138

[59] Kjölvmark C Tschernij E Öberg J Påhlman LI Linder A Åkesson P . Distinguishing asymptomatic bacteriuria from urinary tract infection in the elderly - the use of urine levels of heparin-binding protein and interleukin-6. Diagn Microbiol Infect Dis. 2016;85:243-248. 27039283 10.1016/j.diagmicrobio.2016.03.005

[60] Rodhe N Löfgren S Strindhall J Matussek A Mölstad S . Cytokines in urine in elderly subjects with acute cystitis and asymptomatic bacteriuria. Scand J Prim Health Care. 2009;27:74-79. 19247873 10.1080/02813430902757634 PMC3410465

[61] Sundvall PD Elm M Ulleryd P , et al. Interleukin-6 concentrations in the urine and dipstick analyses were related to bacteriuria but not symptoms in the elderly: a cross sectional study of 421 nursing home residents. BMC Geriatr. 2014;14:88-88. 25117748 10.1186/1471-2318-14-88 PMC4137105

[62] Lacerda Mariano L Ingersoll MA . The immune response to infection in the bladder. Nat Rev Urol. 2020;17:439-458. 32661333 10.1038/s41585-020-0350-8

[63] Hosseini M Ahmadzadeh H Toloui A , et al. The value of interleukin levels in the diagnosis of febrile urinary tract infections in children and adolescents; a systematic review and meta-analysis. J Pediatr Urol. 2022;18:211-223. 35184943 10.1016/j.jpurol.2022.01.010

[64] Beyer AK Currea GCC Holm A . Validity of microscopy for diagnosing urinary tract infection in general practice – a systematic review. Scand J Prim Health Care. 2019;37:373-379. 31304845 10.1080/02813432.2019.1639935 PMC6713105

