# Supplementary material for: What is the Diagnostic Accuracy of Novel Urine Biomarkers for Urinary Tract Infection?
Source: Biomark Insights. 2023 Jan 23;18:11772719221144459. doi: 10.1177/11772719221144459 (PMC9902898; doi:10.1177/11772719221144459)
Supplement: sj-docx-1-bmi-10.1177_11772719221144459 – Supplemental material for What is the Diagnostic Accuracy of Novel Urine Biomarkers for Urinary Tract Infection? [file sj-docx-1-bmi-10.1177_11772719221144459.docx]

| **Study** | **Design** | **Setting; Country** | **Population** | **Participant Conditions (n)** | **Index Tests** | **Reference Standard (threshold)** | **Age range (mean/median)** | **Gender (No (%) Female)** | **Sample Size, prevalence (No, (%) UTI)** | **Author Conclusion** |
| --- | --- | --- | --- | --- | --- | --- | --- | --- | --- | --- |
| OBJECTIVE 1 ONLY | | | | | | | | | | |
| Bai 2018^24^ | Prospective Cohort | OPD: China | Outpatients with suspected UTI | Suspected UTI (253) | MPO:Creatinine | Urine Culture (one or two urinary pathogen at a concentration of ≥10^5^ CFU/ml) | 39-75 (55.8) | 210 (83.0) | 253 (62.1) | MCR higher in positive cultures |
| Benlier 2020^49^ | Case Control | OPD: Turkey | Patients with bladder cancer, and patients with or without UTI presenting to a urology clinic | Bladder cancer (61), acute UTI (30), control (30 | HMGB1 | Urine culture (more than 10^5^ colonies) and dipstick (a positive leukocyte dipstick) | 42.4-79 (59.4) | 37 (30.6) | 121 (50.8) | HMGB1 higher in UTI vs control |
| Burdof 1970^50^ | Case Control | Not given | Hospital patients whose urine was sent to the Bacteriology laboratory for culture with a viable count of <10,000 organisms / ml (control) and >100,000 organisms / ml (UTI) | UTI (12), Controls (9) | IgA excretion/24hrs; IgD excretion/24hrs, IgG excretion/24hrs; IgM excretion/24hrs Siderophilin clearance; Albumin clearance | Urine Culture (>10^5^ organisms/ml in two specimens) | - | 18 (85.7) | 21 (42.9) | Increase in IgA, IgG, siderophilin and albumin clearance in UTI patients |
| Deo 2004^39^ | Case Control | Not given; India | Adult patients showing signs of urinary tract infection | UTI (17), Controls (10) | sIGA | Urine Culture (10^3^-10^5^ organisms / ml or two unrelated species) | - | - | 27 (63.0) | sIgA higher in UTI versus Controls |
| Flores-Figueroa 2017^23^ | Prospective Cohort | IP; Mexico | Catheterised hospital patients with urinary tract infection | Catheter associated UTI (16) | IL-8 | Urine Culture or dipstick (leukocytes >5 x 10(9)/l or a pure/mixed culture of bacteria >10(3) CFU/ml) | 60-98 (78.8) | 11 (68.8) | 16 (100) | IL-8 levels rise early in UTI |
| Forster 2020^51^ | Case Control (secondary analysis of RCT) | OP; USA | Adults patient with spinal cord injury, multiple sclerosis, or spina bifida, who used intermittent catheterisation for bladder managed, had a least two self-diagnosed UTIs in the previous 12 months, and lived in the community | No UTI (29), Unlikely UTI (67) and Likely UTI (8) | NGAL | Symptoms and urine culture (bacterial count ≥ 10^4^ cfu/ml urine) | 26.3-48.7 (37.5) | 8 (29.6) | 27 (7.7) | NGAL levels are associated with likelihood of UTI. |
| Gadalla 2019^32^ | Case Control (secondary analysis of RCT) | GP; UK | Women presenting to primary care with at least one symptoms of UTI (dysuria, urgency, or frequency) | UTI (79), no UTI (104) | IL-1β + MMP9 (combined), MMP9+NGAL+IL-8+IL-1B (combined) | Urine Culture and microscopy (from 10^4^->10^5^ CFU/ml depending on the type and relative proportions of bacteria present, and WBC count) | 18-85 (na) | 183 (100) | 183 (42.1) | Urine biomarkers could be used for POC diagnostics but require further validation. |
| Greenwell 1995^41^ | Case Control | Not given; USA | Adults (no more details given) | Cystitis (15), Pyelonephritis (13), Controls (33) | Urinary Free Secretory Component, Secretary IgA | Symptoms (cystitis = dysuria, frequency, pyelonephritis = as above with fever, flank pain, nausea, and vomiting); urine culture (bacterial count ≥ 10^5^ cfu/ml urine) | - | - | 82 (59.8) | Excretion of sIgA and FSC were increased in the pyelonephritis group, not in the controls or cystitis group. |
| Hu 2016^52^ | Case Control | IP: China | Patients hospitalised with UTI | UTI (73), Controls (112) | N-Nitrosamines (NAms): NDMA, NMEA, NPYR, NDEA, NPIP, NMOR, NDPA, NDBA, NDPhA. All measured in ng/mL | Clinical symptoms, leukocyte esterase activity, nitrite test, urine culture (according to IDSA guidelines) | 21-90 (53.7) | - | 185 (39.5) | Urinary Nams several fold higher in UTI compared to control subjects |
| Jacobson 1998^26^ | Case Control | IP: Sweden | Patients admitted to hospital with non-obstructive pyelonephritis | Acute pyelonephritis (29), controls (12) | IL-1RA, sIL-6 Receptor, IL-6, IL-8, IL-10, G-CSF, sTNFR-1; sTNFR-2 | Symptoms; urine culture; C-reactive protein measurement (fever >38^o^C, flank pain; ≥ 10^5^ cfu/ml urine; CRP ≥ 20) | 16-48 (34.1) | 29 (100) | 29 (70.1) | Urinary levels of sIL-6R were lower in patients than in controls |
| Johnson 2014^53^ | Case Control | Not given; UK | No detail given | UTI (36), Controls (52) | Urinary volatile organic compounds (VOCs) | Urine Culture (no threshold specified) | na (60.9) | 60 (68.2) | 88 (40.9) | Urinary VOCs potential good rapid biomarker UTIs |
| Kjolvmark 2014^33^ | Case Control | IP+GP: Sweden | Patients presenting in primary care with symptoms of UTI, patients presenting to ED with suspected UTI | Definite cystitis (118), probable cystitis (39), definite pyelonephritis (59), probably pyelonephritis (6), no UTI (143), controls (25). | u-HBP, IL-6 | Symptoms (typical symptoms of cystitis, and fever >37.5^o^C, elevated plasma CRP for pyelonephritis); urine culture  (≥10^4^ CFU/ml of *E. coli* or *S.saprophyticus*, 10^5^ CFU/ml of a secondary pathogen) or positive urine dipstick | 18-93 (57.8) | 284 (69.4) | 409 (58.9) | U-HBP was the best diagnostic marker for UTI and could discriminate between cystitis and pyelonephritis. |
| Lam 2014^54^ | Case Control | Not given; Hong Kong | No detail given | Bacterial UTI (88), control (61) | Acetic acid/creatinine, TMA | Microscopy and Urine Culture (1 of: moderate or large blood, positive leukocyte esterase, positive nitrite, RBC ≥52/field,  10^5^ CFU/ml) | 23-104 (67.5) | 60 (40.3) | 149 (59.1) | Acetic acid/creatinine ratio could help diagnose UTI |
| Lussu 2017^55^ | Case control | IP; Italy | No detail given | UTI (72), Controls (61) | Acetic acid/creatinine, TMA | Symptoms urine culture  (dysuria, frequency, urgency; ≥10^3^ cfu/ml urine when associated with clinical significant signs in symptomatic patients) | na, (58.2) | 90 (67.7) | 72 (54.1) | Acetate and Tremethilamine had good diagnostic accuracy for UTI |
| Nishitani 1999^56^ | Case Control | Not given: Japan | Women (no more details given) | Cystitis (18), Controls (18) | Adrenomedullin | Symptoms; urine microscopy and culture (dysuria, frequency, and urgency; 10 or more urine leukocytes per high power field, detection of microorganisms in microscopy) | na, (51.7) | 36 (100) | 36 (50) | Adrenomedullin levels increase in patients with cystitis and return to the control levels after antibiotic treatment |
| Olszyna 1998^37^ | Case Control | Not given: Netherlands | No detail given | Urosepsis (28), Controls (22) | (sTNFR) types I and II, IL-1B, IL-1ra, sIL-1RII, IL-10, TNF-a | Urine Culture | - | - | 50 (60) | sTNFR increased in urosepsis compared with controls. |
| Olszyna 2000^28^ | Case Control | Not given: Netherlands | Patients with suspected gram-negative urosepsis | Urosepsis (33), Controls (16) | IL-8, (GRO)–a, (ENA)–78 | Symptoms; signs of sepsis; pyuria; gram -ve bacteria in urine (>100 cells per mm^3^) | - | - | 64 (51.6) | Increased IL-8, GRO-a, and ENA-78 in urosepsis |
| Olszyna 2001^25^ | Prospective Cohort | IP: Netherlands | Catheterised patients undergoing major abdominal surgery | UTI (10), controls (20) | IL-6; IL-8 | Symptom and Urine Culture (>10^5^ CFU/ml and <3 bacterial species/ml) | na, (65.3) | 19 (63.3) | 10 (33.3) | In post-op patients who develop UTI, IL-8 but not IL-6, marks the early phase of the local host response to the infection |
| Price 2017^57^ | Case Control | IP:USA | Women with symptoms indicative of a UTI | UTI (50), Controls (50) | NGAL | Symptoms and Urine Culture (≥10^5^ organisms/ml | 19.3-80.1 (51.2) | 100 (100) | 100 (50) |  |
| Pupek-Musialik 1990^58^ | Case Control | Not given; Poland | No detail given | UTI (25), Pyelonephritis (25), Controls (25) | B2M | Culture and microscopy (≥10^5^ CFU/ml) | - | 40 (53%) | 75 (33%) | No difference in B2M between cystitis patients and healthy controls, but acute pyelonephritis patients have increased B2M in urine. |
| Sahin 2015^59^ | Case Control | Not given: Turkey | Pregnant women | UTI (25), not UTI (25), Controls (25) - all pregnant | MPO | Urine Culture  (≥10^5^ CFU/ml of one or two pathogens) | 24.5-29.2 (27.3) | 75 (100) | 75 (66.7) | MPO higher in UTI compared with controls but not in contaminated samples |
| Sandberg 1986^60^ | Case Control | Not given; Sweden | No detail given | Pyelonephritis (101), Cystitis (32), Fever of non-renal origin (12) | NAG and AAP | Symptoms (pyelonephritis only) and urine culture (≥10^5^ CFU/ml of one species) | 17-89 (48.4) | 142 (97.9) | 145 (91.7) | NAG and AAP higher in pyelonephritis than cystitis. Cystitis and fever of non-renal origin were comparable |
| Short 1987^38^ | Case Control | Not given: USA | Catheterised women with recurrent cystitis | Recurrent cysitis with UTI (13), Recurrent cystitis without UTI (39), Controls (20) | sIgA, IgA, IgG | Urine Culture (≥10^2^ colonies/ml) | 26-42 (na) | 25 (100) | 25 (20.0) | Significant increase in IgA and sIgA in UTI versus non-UTI or control samples |
| Tyagi 2016^31^ | Case Control | OPD: Taiwan | Patients routinely visiting a urology clinic | UTI (62), OAB (59), Control (26) | GRO-alpha (CXCL-1); IL-1 beta, IL-1RA, IL-8; CXCL-10, MCP-1, NGF, PDGF | Symptoms; urine culture; positive dipstick (urgency and burning sensation on urination; no thresholds given) | na, (52.0) | 121 (82.3) | 147 (41.5) | CXCL-1, CXCL-8, CXCL-10, were elevated in UTI patients. CXCL-1 was the most specific (ROC curve) and was a predictor for UTI |
| Vera 2018^61^ | Case Control | Not given: USA | Women with interstitial cystitis with and without Hunner’s lesions, men with bacterial and radiation cystitis | Cystitis, Radiation cystitis, control | MIF, MIF:creatinine | Urine Culture (no threshold given) | 61.2-69.2 (64.2) | 0 (0) | 197 (25.4) | Urinary MIF is elevated in patients with bladder inflammation |
| Wu 2019^35^ | Case Control | IP: China | Urine samples collected in a hospital | UTI (157), no UTI (61), control (40) | Urinary HBP, IL-6 | Urine Culture  (no threshold given) | 19-87 (na) | 129 (50) | 157 (60.9) | Urine IL6 measurements are not significantly different between UTI and non UTI. Urine HBP and white cell count are increased in UTI |
| Zhu 2016^62^ | Case Control | IP: China | Patients with primary urothelial cell carcinoma | Cystitis (42), Bladder Cancer (92), Controls (38) | C-X-C motif chemokine ligand 5 (CXCL5)/Creatinine | Urine Culture; urine leukocytes  (no thresholds given) | 25-87 (56.6) | 59 (34.3) | 172 (24.4) | CXCL5 could be a marker for bladder cancer. |
| **BOTH OBJECTVIVES 1 AND 2** | | | | | | | | | | |
| Ciszek 2006^30^ | Case Control | Not given: Poland | Patients with stable graft function 12-60 months after kidney transplantation on routine ambulatory care visit. 8 additional UTI patients recruited on admission to hospital | UTI (22), ASB (13), Controls (25) | IL-6; IL-8 | Urine Culture (≥10^5^ CFU/ml of one bacterial species) | - | - | 60 (21.7) | Increase in IL-8 but not IL-6 in ASB versus no growth, after kidney transplant |
| Determann 2007^42^ | Case Control | ED; Netherlands | Not specified | Upper UTI (15), Lower UTI (55), ASB (9), Control (10) | sTREM-1 | Symptoms; presence of urine leukocytes; urine culture (≥10^5^ CFU/ml) | - | - | 89 (78.7) | Limited diagnostic value of urinary sTREM-1 in patients with suspected UTI. |
| Ethel 2006^40^ | Case Control | Not given; India | Women (no more details given) | UTI (30), ASB (30), Controls (30) | sIgA, IgA, IgG and IgM binding to mixed coliform antigen and clinical isolate antigen | Symptoms; bacterial count ≥ 10^5^ cfu/ml urine) | 18-43 (26a) | 90 (100) | 90 (33.3) | High levels of IgG in UTI and ASB versus controls. IgM lower in UTI than ASB. Increase in sIgA in UTI |
| Hedges 1992^45^ | Case Control | Not given; Sweden | Non-pregnant women with asymptomatic bacteriuria, hospitalised patients with pyelonephritis, non-pregnant controls | Pyelonephritis (29), ASB (22 women, 42 episodes), Controls (not given) | IL-6 | Symptoms (fever ≥38^o^C, flank pain, costovertebral angle tenderness); Urine Culture (≥10^5^ CFU/ml) | - | 61 (100) | 61 (47.5) | Increase in IL-6 in UTI group |
| Jacobson 1994^27^ | Case Control | IP: Sweden | Patients admitted to hospital with pyelonephritis | Acute pyelonephritis (43), (from the same group at different time cystitis (8), ASB (8)), Control (37) | IL-6; IL-8 | Symptoms (fever <38^O^C, flank pain),  Urine culture (≥ 10^5^ cfu/ml urine); CRP (≥ 20) | - | 80 (100) | 80 (53.8) | Patients with acute E Coli pyelonephritis have increased concentrations of IL-6 and IL-8 in serum and in urine. |
| Jacobson 1996^36^ | Case Control | IP: Sweden | Patients admitted to hospital with non-obstructive pyelonephritis | Acute pyelonephritis (41), (from the same group at different time cystitis 6), ASB (6)), Control (26) | sTNFR-1; sTNFR-2; IL-1 | Symptoms (fever <38^O^C, flank pain),  Urine culture (≥ 10^5^ cfu/ml urine); CRP (≥ 20) | 16-70 (33.9) | 67 (100) | 41 (61.2) | Patients with acute pyelonephritis had higher concentrations of sTNRF 1 and sTNRF II in both urine and serum. IL-1ra was significantly lower compared to controls. |
| Kjolvmark 2016^34^ | Case Control | NH: Sweden | Nursing home residents | UTI (49), Indwelling catheter (18), ASB (38), no UTI (57) | u-HBP, IL-6 | Symptoms (dysuria, frequency, urgency, suprapubic pain, heamturia, and fever >37.5^o^C, elevated plasma CRP for pyelonephritis); urine culture  (>10^5^ CFU/ml of a single organism) | 80-93 (86.8) | 108 (66.7) | 162 (30.2) | IL-6 is discriminatory between UTI and ASB and controls, u-HBP is not |
| Rodhe 2009^29^ | Case Control | GP; Sweden | Patients aged 80 and above | UTI (16), ASB (24), Controls (20) | CCL2 (MCP-1), CXCL1 (GRO-a), IL-1B, IL-6, IL-8 (CXCL8), IL-10, IL-12, IL-18, and TNF-a | Symptoms (new onset of dysuria, frequency and/or urgency), significant bacterial growth (≥10^8^ CFU/l of *E.coli* in two consecutive samples) | ≥80 (85.1) | 52 (86.7) | 60 (26.7) | Higher urinary levels of CXCL1, IL-8, and IL-6 in patients with acute cystitis than in patients with ASB |
| Sundvall 2014^44^ | Cross-sectional | NH: Sweden | Nursing home residents | Positive urine culture (135), negative urine culture (286) | IL-6 | Urine Culture (≥10^5^ CFU/ml, or, ≥10^3^ CFU/ml of *E.coli*, *Entercoccus* faecalis, or kledisella. | 63-100 (86.4) | 291 (68.5) | 421 (0) | IL-6 not useful in determining ASB vs symptomatic UTI in NH residents |
| **OBJECTIVE 2 ONLY** | | | | | | | | | | |
| Sunden 2016^22^ | Prospective Cohort | Not given; Sweden | Nursing home residents | ASB (35), UTI (22 in 13 ASB patients). 8 male patients had a permanent indwelling catheter. | IL-6; IL-8 | Symptoms; urine culture (≥10^5^ CFU/ml or the same uropathogen in two consecutive samples (for ASB)). | 54-99 (82) | 20 (57.1) | 35 (37.1) | IL-6, not IL-8, sig diff between UTI, ASB and Control |

**Supplementary Table 1. Summary of included studies.**
