# Supplementary material for: What is the Diagnostic Accuracy of Novel Urine Biomarkers for Urinary Tract Infection?
Source: Biomark Insights. 2023 Jan 23;18:11772719221144459. doi: 10.1177/11772719221144459 (PMC9902898; doi:10.1177/11772719221144459)
Supplement: sj-docx-4-bmi-10.1177_11772719221144459 – Supplemental material for What is the Diagnostic Accuracy of Novel Urine Biomarkers for Urinary Tract Infection? [file sj-docx-4-bmi-10.1177_11772719221144459.docx]

| Reference | Biomarker | Threshold used | *n* | Prevalence | Sensitvity | Specificity | PPV | NPV | LR+ve | LR-ve | Area under ROC | 95% limits of Area under ROC |
| --- | --- | --- | --- | --- | --- | --- | --- | --- | --- | --- | --- | --- |
| **Urinary Tract Infection versus no Urinary Tract Infection^1^** | | | | | | | | | | | | |
| Lam 2014 | Acetic Acid:Creatinine ratio | 0.03mmol/mmol | 149 | 0.59 | 0.91 | 0.95 | 0.96 | 0.88 | 17.00 | 0.10 | - | - |
| Gadalla 2019* | IL-1B + MMP9 | not given | 55 | 0.43 | 0.67 | 0.76 | 0.68 | 0.75 | 2.80 | 0.44 | 0.82 | 0.69-0.94 |
| Gadalla 2019* | IL-1B + MMP9 + NGAL + IL-8 | not given | 55 | 0.43 | 0.64 | 0.89 | 0.82 | 0.76 | 6.09 | 0.40 | 0.81 | 0.68-0.94 |
| Kjolvmark 2014 | IL-6 | 30pg/ml | 320 | 0.55 | 0.52 | 0.93 | 0.89 | 0.65 | 7.32 | 0.52 | - | - |
| Flores-Figueroa 2017† | IL-8 | 100pg/ml | 16 | 0.69 | 0.94 | 0.88 | 0.88 | 0.99 | 7.50 | 0.07 | - | - |
| Bai 2018† | MCR | 194ng/g | 253 | 0.62 | 0.66 | 0.95 | 0.95 | 0.63 | 13.20 | 0.36 | - | - |
| Kjolvmark 2014 | U-HBP | 30ng/ml | 320 | 0.55 | 0.89 | 0.90 | 0.90 | 0.89 | 8.75 | 0.12 | - | - |
| **Urinary Tract Infection versus Healthy Controls** | | | | | | | | | | | | |
| Lussu 2017 | Acetate | not given | 112 | 0.46 | - | - | - | - | - | - | 0.92 | 0.81-1.0 |
| Benlier 2020 | HMGB1 | not given | 60 | 0.50 | 0.77 | 0.67 | 0.70 | 0.74 | 2.32 | 0.35 | 0.71 | 0.58-0.85 |
| Lussu 2017 | Trimethylamine | not given | 112 | 0.46 | - | - | - | - | - | - | 0.94 | 0.86-1.0 |
| Johnson 2014 | Urinary volatile organic compounds (VOCs) | not given | 88 | 0.41 | 0.81 | 0.98 | 0.97 | 0.88 | 40.00 | 0.20 | - | - |
| **Urinary Tract Infection versus mixed non-infective conditions^2^** | | | | | | | | | | | | |
| Tyagi 2016~ | CXCL-10 | not given | 147 | 0.41 | 0.38 | 0.63 | 0.42 | 0.59 | 1.03 | 0.98 | 0.54 | - |
| Tyagi 2016~ | GRO-a (CXCL1) | 20pg/ml | 147 | 0.41 | 0.60 | 0.81 | 0.69 | 0.74 | 3.10 | 0.50 | 0.71 |  |
| Tyagi 2016~ | Il-8 | 20pg/ml | 147 | 0.41 | 0.65 | 0.71 | 0.61 | 0.74 | 2.25 | 0.49 | 0.71 | - |
| **Urinary Tract Infection versus Asymptomatic bacteruria^3^** | | | | | | | | | | | | |
| Rodhe 2009 | GRO-a (CXCL1) | 150pg/mg creatinine | 40 | 0.40 | 0.69 | 0.79 | 0.69 | 0.79 | 3.29 | 0.39 | - | - |
| Kjolvmark 2016 | IL-6 | 30pg/ml | 87 | 0.56 | 0.59 | 0.84 | 0.67 | 0.76 | 3.69 | 0.49 | - | - |
| Rodhe 2009 | IL-6 | 30pg/mg creatinine | 40 | 0.40 | 0.81 | 0.96 | 0.93 | 0.88 | 20.00 | 0.20 | - | - |
| Rodhe 2009 | IL-8 | 135pg/mg creatinine | 40 | 0.40 | 1.00 | 0.71 | 0.70 | 1.00 | 3.45 | 0.00 | - | - |
| Kjolvmark 2016 | U-HBP | 30ng/ml | 87 | 0.56 | 0.96 | 0.33 | 0.48 | 0.93 | 1.43 | 0.12 | - | - |
| Determann 2007 | Urinary sTREM-1 | not given | 79 | 0.89 | 0.19 | 0.89 | 0.93 | 0.12 | 1.73 | 0.91 | - | - |

**Supplemantary Table 3. Diagnostic accuracy data.**

All studies case control unless indicated: † - Prospective Cohort; * - Restrospective Cohort

1. No Urinary Tract Infection refers to recruited participants who had symptoms or suspicion of UTI but no infection identified with the reference standard.

2. Mixed non-infective conditions: participants in this comparison group included healthy controls and patients with overactive bladder syndrome but no current UTI.

3: Assesses diagnostic accuracy of the biomarker to differentiate symptomatic from asymptomatic bacteriuria assuming symptoms are unknown.
